# Honokiol attenuates diabetic nephropathy by targeting SIRT3 to suppress mitochondrial ROS-induced pyroptosis

**DOI:** 10.1186/s13098-025-02045-4

**Published:** 2025-12-02

**Authors:** Ke Yu, Maodong Liu, Tao Zhang, Canghui Guo, Lingyu Du, Min Li, Qian Wang, Ning Han, Yanqing Chi, Ying Li

**Affiliations:** 1 Department of Nephrology, Hebei Medical University Third Hospital, No. 139, Ziqiang Road, Shijiazhuang, 050051 Hebei Province China; 2 Hebei Key Laboratory of Diabetic Kidney Disease, Shijiazhuang, 050051 China

**Keywords:** Honokiol, Diabetic nephropathy, Pyroptosis, SIRT3, Mitochondria

## Abstract

**Background:**

Diabetic nephropathy (DN) persists as the leading cause of end-stage renal disease worldwide. Growing evidence indicates that mitochondrial dysfunction triggers NLRP3 inflammasome activation and subsequent pyroptosis, which play crucial roles in DN development. Honokiol (HKL), a natural compound with the ability to upregulate SIRT3 expression, shows promise in protecting mitochondrial function. This study investigates HKL’s renoprotective effects in DN and explores its mechanism of action through the SIRT3-mediated regulation of mitochondrial ROS, NLRP3 inflammasome, and pyroptosis.

**Materials and methods:**

db/db diabetic mice and HK-2 cells subjected to hyperglycemic stimulation were used to assess the therapeutic effects of HKL. Renal function, pyroptosis, and mitochondrial homeostasis were assessed via biochemical assays, histopathological and immunohistochemical analyses, transmission electron microscopy, Western blotting, and immunofluorescence staining. SIRT3 overexpression and knockdown were performed to validate its regulatory role, while the mtROS scavenger MitoTEMPO was utilized to confirm the pivotal involvement of mtROS in the pyroptotic pathway.

**Results:**

HKL treatment significantly ameliorated renal dysfunction and pathological damage in diabetic mice. Mechanistically, HKL upregulated SIRT3 expression, thereby improving mitochondrial function (maintaining structural integrity, stabilizing the membrane potential, and reducing mtROS accumulation), which in turn suppressed NLRP3 inflammasome activation and subsequent GSDMD-mediated pyroptosis. SIRT3 overexpression mimicked the protective effects of HKL, whereas SIRT3 knockdown attenuated its efficacy, confirming the essential role of SIRT3 in this process. Furthermore, mtROS scavenging by MitoTEMPO mitigated pyroptosis, reinforcing the dependence of the effects of HKL on the SIRT3-mtROS axis.

**Conclusion:**

By upregulating SIRT3 expression to maintain mitochondrial homeostasis and suppress mtROS-NLRP3-mediated pyroptosis, HKL emerges as a promising therapeutic strategy for DN.

**Graphical abstract:**

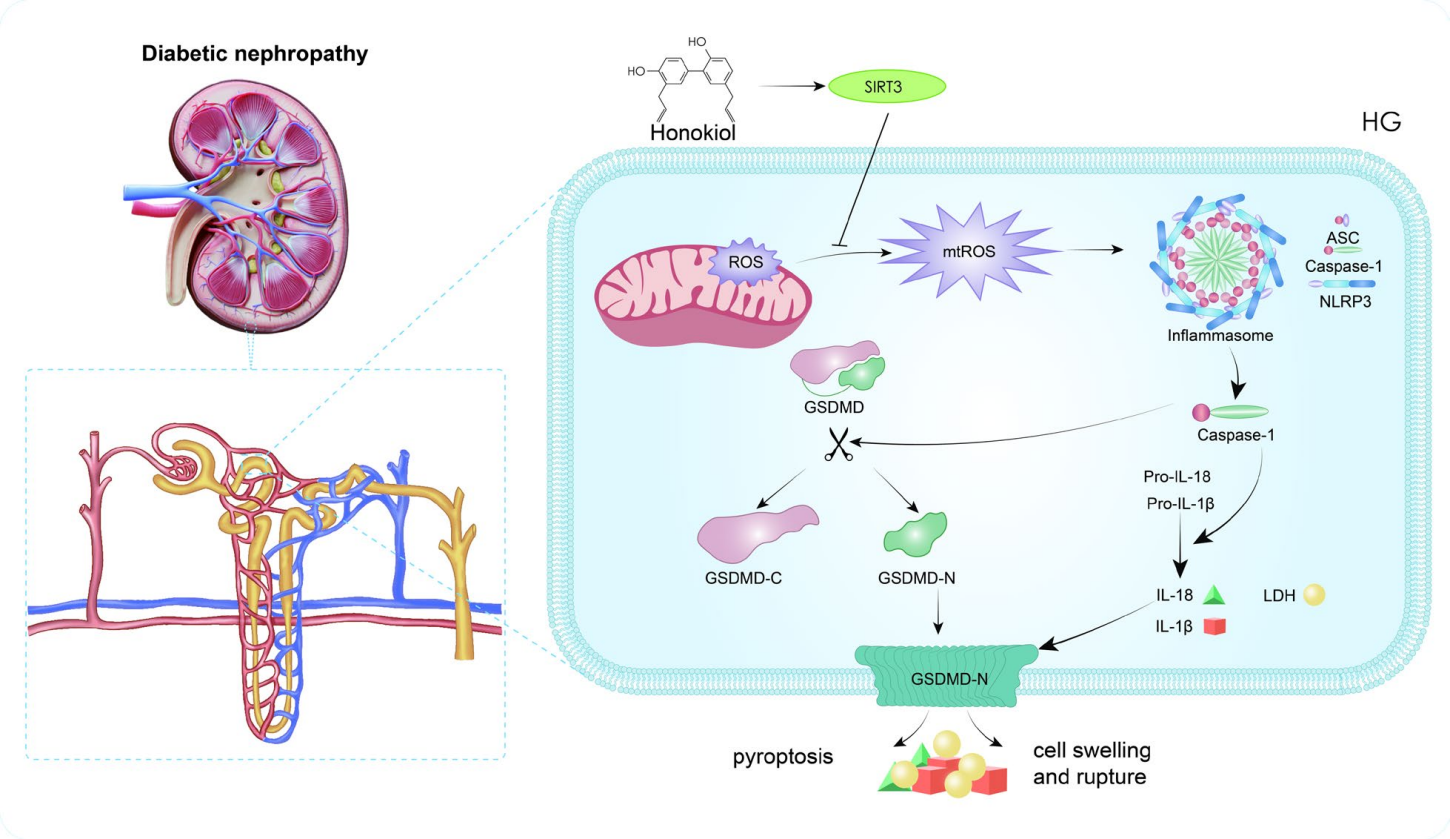

**Supplementary Information:**

The online version contains supplementary material available at 10.1186/s13098-025-02045-4.

## Introduction

Diabetes mellitus and its associated complications have emerged as critical global public health challenges. Recent epidemiological statistics from the International Diabetes Federation revealed that, in 2021, there were 537 million cases of diabetes worldwide, with forecasts suggesting an increase to 784 million by 2045 [1]. Among the most severe microvascular complications, diabetic nephropathy (DN) has shown a parallel increase in incidence, ultimately affecting approximately 40% of all diabetic patients [2].

Current therapeutic strategies for DN, including inhibitors of the renin‒angiotensin system (RAS), sodium‒glucose cotransporter-2 (SGLT2) inhibitors, and novel nonsteroidal mineralocorticoid receptor antagonists (MRAs), demonstrate partial efficacy in attenuating the progression of renal pathology. However, these treatments fail to completely halt renal function deterioration, particularly in advanced-stage DN [3], which places patients at heightened risk for swift progression of the disease and mortality associated with cardiovascular issues [4, 5]. Notably, DN remains the primary contributor to chronic kidney disease (CKD) and end-stage renal disease (ESRD) worldwide [6]. Between 2000 and 2015, the global prevalence of diabetes-related ESRD surged from 19.0% to 29.7%, while the proportion of new ESRD cases caused by diabetes expanded from 22.1% to 31.3% [7], imposing an escalating burden on healthcare systems. Given the limited therapeutic options and the substantial socioeconomic impact of DN, further research into its underlying mechanisms and novel interventions is urgently needed.

Recent research has emphasized the significant involvement of inflammatory responses in DN pathogenesis, with a particular focus on NLRP3 inflammasome-triggered pyroptosis as a pivotal pathological contributor [8, 9]. This pathway operates through a well-defined cascade: first, caspase-1 activation mediates cleavage of Gasdermin D (GSDMD), generating its membrane-pore-forming N-terminal domain (GSDMD-N); the ensuing disruption of the membrane allows for the release of key inflammatory mediators, including IL-18 and IL-1β [10], thereby establishing a vicious “inflammation‒injury” cycle. Consequently, targeting the modulation of the NLRP3 inflammasome represents a potentially effective treatment strategy for DN [11, 12].

Experimental evidence indicates that mitochondrial impairment acts as a key initiator of NLRP3 inflammasome signalling. Mitochondria play pivotal roles in the generation of energy and the synthesis of reactive oxygen species (ROS). In the event of mitochondrial damage, there is increased release of ROS, which has been demonstrated to directly promote the assembly of the NLRP3 inflammasome [13]. Among the regulators of mitochondrial homeostasis, sirtuin 3 (SIRT3), the major mitochondrial deacetylase, has drawn considerable interest for its role in preserving metabolic equilibrium by modulating the acetylation status of key enzymes [14]. Accumulating evidence links SIRT3 dysfunction to the pathogenesis of various metabolic diseases, particularly diabetes and associated complications [15–17]. Importantly, SIRT3 exerts protective effects against DN through multiple mechanisms, including attenuation of oxidative stress and regulation of energy metabolism [18]. By maintaining mitochondrial fitness and scavenging excess mtROS, the upregulation and restored activity of SIRT3 may thus attenuate a primary trigger for NLRP3 inflammasome assembly. Therefore, enhancing SIRT3 function presents a rational strategy to mitigate NLRP3-dependent inflammation and pyroptosis in DN.

Building on these insights, we investigated honokiol (HKL, C₁₈H₁₈O₂, CAS: 35354-74-6), a bioactive substance derived from *Magnolia officinalis*, has garnered significant attention for its pleiotropic therapeutic properties. Modern pharmacological studies have established its potent antioxidant and anti-inflammatory effects [19–21]. Critically, HKL demonstrates specific SIRT3 upregulatory capacity and confers robust mitochondrial protection, positioning it as a promising modulator of metabolic and inflammatory pathways [22, 23]. Its potential in DN is particularly promising when compared to broad-spectrum antioxidants or other compounds known to enhance SIRT3 expression or activity (e.g., resveratrol); HKL exhibits a unique combination of mitochondrial stabilization and efficient SIRT3 enhancement, a mechanism that has not been fully explored in the context of DN. HKL has shown therapeutic efficacy against multiple renal pathologies, including fibrosis, ischaemia‒reperfusion injury, and acute kidney injury [24–27]. On the basis of this evidence, we propose a novel hypothesis: HKL may mitigate DN progression through regulation of the SIRT3-mediated mtROS‒NLRP3 signalling pathway, thereby suppressing pyroptosis. **T**o our knowledge, this is the first study to systematically investigate the role of HKL in targeting the SIRT3-mtROS-NLRP3 axis for DN therapy. This study enhances our understanding of the pathogenesis of DN while simultaneously offering a theoretical foundation for developing innovative therapies targeting the mitochondria‒inflammation network.

## Materials and methods

### Clinical specimens

Renal tissue samples, including renal biopsy samples from 10 patients diagnosed with DN (case group) and adjacent normal renal tissues from 10 patients who had undergone radical nephrectomy for renal tumors (control group), were obtained from Hebei Medical University Third Hospital. The Ethics Committee of Hebei Medical University Third Hospital formally approved all study protocols (Approval No. W2023-024-1), with documented informed consent obtained prior to all tissue acquisitions.

### Experimental animals

Male C57BL/6Ks db/db mice (6 weeks old, 34.5 ± 1.9 g) and db/m mice (21.4 ± 1.3 g) were acquired from Huachuang Xinno Pharmaceutical Technology Co., Ltd., located in Jiangsu, China. All mice were housed in individually ventilated cages with a maximum of 5 mice per cage, provided with standard laboratory chow and water ad libitum. Following a two-week acclimatization period in a specific pathogen-free environment (temperature: 22 ± 1 °C, humidity: 50 ± 10%, light/dark cycle: 12 h each), db/db mice exhibiting a random tail vein blood glucose ≥ 16.7 mmol/L on consecutive days were randomly assigned to either the model group (*n* = 10) or the HKL-treated group (*n* = 10), while db/m mice served as controls (*n* = 10). The HKL-treated group underwent daily intraperitoneal injections of 5 mg/kg HKL (dissolved in 2% DMSO, 10% PEG300, 88% saline) for 8 weeks, while control and model groups received vehicle alone on the same schedule. Approval for all animal procedures was granted by the Experimental Animal Welfare Ethics Committee of Hebei Medical University (IACUC-Hebmu-2023019).

### Cell culture and treatment

Human renal proximal tubular epithelial cells (HK-2, ATCC, USA) were cultured in DMEM/F12 (1:1) medium (Gibco, USA) supplemented with 10% fetal bovine serum (CellMax, China) and 1% penicillin‒streptomycin (Gibco, USA). HK-2 cells were maintained under standard culture conditions (37 °C, 5% CO₂, humidified atmosphere) and were routinely passaged at 80–90% confluence using 0.25% trypsin-EDTA (Gibco, USA). Cells between passages 3 and 10 were used for all experiments. For the study, cells were randomly allocated to four treatment groups for experimental analysis: (1) normal glucose control (NG, 5.5 mM glucose); (2) NG plus HKL treatment (NG + HKL, 5.5 mM glucose + 10 µM HKL); (3) high-glucose model (HG, 30 mM glucose); and (4) HG plus HKL treatment (HG + HKL, 30 mM glucose + 10 µM HKL). HKL was prepared as a 100 mM stock solution in DMSO and diluted in culture medium to a final working concentration of 10 µM, with the DMSO concentration not exceeding 0.1% in all groups. Following 48 h of incubation under standard culture conditions, the cells were processed for subsequent procedures.

For the transfection experiments, four additional groups were established: (1) high glucose plus empty vector control (HG + C, transfected with pcDNA3.1); (2) high glucose plus SIRT3 overexpression (HG + OE, transfected with SIRT3 pcDNA); (3) high glucose plus negative control siRNA (HG + siNC, transfected with scrambled siRNA); and (4) high glucose plus SIRT3 knockdown (HG + siSIRT3, transfected with SIRT3 siRNA). Lipofectamine 3000 (Invitrogen, USA) was employed following the supplier’s instructions when the cells reached 40–50% confluence. Following a 6-hour cultivation period in serum- and antibiotic-free media, the cells were maintained in the respective treatment media for another 48 h before immunofluorescence examination or protein isolation for Western blotting.

### Main reagents and antibodies

Honokiol (HKL, HY-N0003; purity ≥ 99.85%) was obtained from MedChemExpress (USA). This compound was isolated from *Magnolia officinalis* through ethanol extraction and subsequent column chromatography purification. Structural characterization of HKL by ¹H NMR spectroscopy and UPLC‒MS analyses is provided in Supplementary Fig. 1. Renal function assay kits for creatinine (Scr, C011-2-1), urinary protein (UPRO, C035-2-1), blood urea nitrogen (BUN, C013-1-1), and albumin (ALB, A028-2-1) were acquired from Nanjing Jiancheng Bioengineering Institute (Nanjing, China). Primary antibodies, including NLRP3 (68102-1-Ig), ASC (10500-1-AP), GSDMD (20770-1-AP), caspase-1 (22915-1-AP), IL-1β (26048-1-AP), IL-18 (10663-1-AP), SIRT3 (10099-1-AP) and β-actin (20536-1-AP) were purchased from Proteintech (Wuhan, China); GSDMD-N (YT7991) was purchased from Immunoway (Suzhou, China); and GSDMD-N (ab215203) was purchased from Abcam (USA).

### Electron microscopy analysis

Renal cortical tissues (1 mm³) were immediately isolated from harvested kidneys and immersed in 4 °C 4% glutaraldehyde for primary fixation. Following buffer washing, the tissue blocks were subjected to secondary fixation in 1% osmium tetroxide for 2 h and then sequentially processed through graded ethanol dehydration, epoxy resin embedding, and ultrathin sectioning. The sections were finally double-stained with uranyl acetate and lead citrate for transmission electron microscopy (TEM) examination. For HK-2 cells, after trypsinization and centrifugation, the cell pellets were primarily fixed with glutaraldehyde and subsequently processed following the same protocol used for renal tissues.

### Histopathological and immunohistochemical analysis

Renal tissues were fixed in 10% neutral buffered formalin for 48 h at 4 °C, followed by standard processing, including dehydration with graded ethanol, xylene clearing, and paraffin embedding. Serial Sects. (2–3 μm thick) were prepared and baked at 60 °C for dewaxing before histological staining with hematoxylin and eosin (HE), Masson’s trichrome, and periodic acid-Schiff (PAS) for comprehensive pathological evaluation.

For immunohistochemistry, the paraffin-embedded sections were subjected to antigen retrieval via EDTA buffer (pH 9.0) and high-pressure heating for 7 min. To inhibit endogenous peroxidase activity, a solution of 3% hydrogen peroxide was applied, which was subsequently followed by a blocking step using 10% normal goat serum at 37 °C for 30 min. The paraffin sections were then incubated overnight at 4 °C with various primary antibodies, including antibodies against ASC (1:200), NLRP3 (1:200), GSDMD-N (1:200), caspase-1 (1:200), SIRT3 (1:400), IL-18 (1:200), and IL-1β (1:200). After thorough washing, the sections were exposed to secondary antibodies obtained from ZSGB-BIO (Beijing, China) at 37 °C for 30 min. Visualization of immunoreactivity was accomplished via a DAB detection kit from ZSGB-BIO. Microscopic imaging was performed via an Olympus BX71 microscope (Tokyo, Japan) and subsequently analysed quantitatively using ImageJ software (NIH).

### Immunofluorescence staining

HK-2 cells were cultured on sterile coverslips placed in 6-well culture plates. Cellular samples were subjected to sequential treatments: initial fixation with 4% paraformaldehyde solution (RT, 30 min), membrane permeabilization with 0.3% Triton X-100/PBS (10 min), and blocking with 10% goat serum (37 °C incubation, 30 min). Paraffin-embedded tissue sections were dewaxed in xylene, rehydrated through a graded ethanol series, and subjected to antigen retrieval via EDTA buffer (pH 9.0) under high-pressure heating.

All samples were incubated overnight at 4 °C with the following primary antibodies: anti-ASC (1:200), anti-NLRP3 (1:100), anti-GSDMD-N (1:200), anti-SIRT3 (1:200), and anti-TOM20 (1:200). After washing, the samples were incubated with either FITC-conjugated (green fluorescence) or TRITC-conjugated (red fluorescence) secondary antibodies at 37 °C for 2 h, followed by nuclear counterstaining with DAPI for 5 min. Fluorescence images were acquired with an Olympus BX71 fluorescence microscope (Japan).

### Western blot analysis

Protein extracts from both renal tissues and HK-2 cells were obtained with RIPA buffer (BestBio, China) and quantified via a BCA assay (Solarbio, China). After SDS‒PAGE separation, the proteins were electrotransferred to PVDF membranes (Millipore, USA). The membranes were blocked with 5% nonfat milk in TBST (37 °C, 1 h) before overnight incubation (4 °C) with primary antibodies against the following proteins: ASC (1:2000), NLRP3 (1:1000), GSDMD-N (1:1000), GSDMD (1:2000), caspase-1 (1:2000), SIRT3 (1:1000), IL-18 (1:2000), IL-1β (1:2000), and β-actin (1:2000). HRP-conjugated goat anti-rabbit/anti-mouse IgG secondary antibody (1:10000) was used for incubation (37 °C, 1 h). The protein bands were detected via enhanced chemiluminescence (ECL) reagents (NCM Biotech, China) and quantified with ImageJ (NIH) after imaging on a Tanon 4800 system.

### Cell viability assay

HK-2 cells were plated in 96-well plates (24 h preculture) and treated with HKL (0–50 µM) under NG (5.5 mM) or HG (30 mM) conditions for 48 h. After adding 10 µL of CCK-8 reagent (MedChemExpress, USA) per well, the plates were incubated at 37 °C for 2 h in the dark. Subsequent spectrophotometric analysis was used to measure the optical density at a wavelength of 450 nm. The experimental group viability percentages were normalized to those of the control groups.

### Mitochondrial membrane potential assay

JC-1 fluorescence staining (Beyotime, C2003S) was used to evaluate changes in the mitochondrial membrane potential (ΔΨm) in treated HK-2 cells. Following 48 h of experimental treatment, the cells were incubated with JC-1 mitochondrial probe stain (37 °C, 30 min, dark conditions) and washed three times with chilled buffer. Fluorescence images (red/green emission) were acquired immediately via a Leica confocal microscope, with subsequent quantitative analysis of intensity ratios performed via ImageJ.

### Mitochondrial morphology analysis

Mitochondrial morphology was assessed via the MitoTracker Red CMXRos fluorescent probe (C1035, Beyotime, China). Treated HK-2 cells were incubated with 200 nM MitoTracker Red working solution for 30 min at 37 °C in the dark. After washing with PBS, live-cell imaging was performed immediately via a Leica confocal microscopy system to capture the mitochondrial architecture.

### Reactive oxygen species (ROS) detection

Mitochondrial superoxide levels, a major component of mitochondrial ROS, were specifically detected using the MitoSOX Red mitochondrial superoxide indicator (M36007; Invitrogen, USA). The cells were treated with 500 nM MitoSOX Red working solution under identical incubation conditions for 30 min and then washed three times with prewarmed (37 °C) Hank’s balanced salt solution (HBSS). To assess intracellular ROS levels, a separate assay was performed using the 2’,7’-dichlorodihydrofluorescein diacetate (DCFH-DA) fluorescent probe (S0033S, Beyotime, China). HK-2 cells were loaded with 10 µM DCFH-DA in serum-free medium at 37 °C under a 5% CO₂ atmosphere for 20 min in the dark, followed by thorough washes with serum-free medium. The fluorescence signals were captured with a Leica confocal microscope and quantitatively analysed via ImageJ software (NIH).

### TUNEL assay for cell death detection

HK-2 cells were analysed for programmed cell death via a TUNEL assay (C1086, Beyotime, China). Briefly, HK-2 cells cultured in 6-well plates were fixed with 4% paraformaldehyde at room temperature for 30 min, followed by washing with PBS. After permeabilization with 0.3% Triton X-100 for 5 min, 50 µL of TUNEL reaction mixture containing terminal deoxynucleotidyl transferase and fluorescein-dUTP was added to each well and incubated at 37 °C for 60 min in the dark. After DAPI counterstaining. Fluorescence images were acquired via a fluorescence microscope (Olympus, Japan) and quantified with ImageJ software to calculate the cell death ratio (TUNEL-positive cells/DAPI-positive cells × 100%).

### Lactate dehydrogenase (LDH) release assay

HK-2 cells were seeded in 6-well plates and treated as indicated. The culture supernatants were collected and clarified via centrifugation (400×g, 5 min). Following the manufacturer’s protocol for the LDH Release Detection Kit (C0019S, Beyotime, China), the supernatant was mixed with assay buffer, 100 µL of LDH detection working solution was added, and the mixture was incubated at 37 °C for 30 min in the dark. The absorbance at 450 nm was measured, and the concentrations were determined against a standard curve.

### ELISA detection of inflammatory cytokines

The concentrations of IL-18 (KE00193) and IL-1β (KE00021) in the cell culture supernatants were determined via ELISA kits (Proteintech). Following the standard protocol, standards or samples were incubated in precoated wells at 37 °C for 2 h. After sequential incubations with detection antibody (1 h) and streptavidin-HRP (40 min), the enzymatic reaction was developed using TMB substrate (incubated at 37 °C in the dark for 15 min) and terminated with stop solution. The absorbance was measured at a wavelength of 450 nm, and the concentrations of the cytokines were then calculated on the basis of standard curves.

### Statistical analysis

Statistical analysis was carried out with GraphPad Prism 10.0 (GraphPad Software, USA), and the results are expressed as the means ± SDs, with the sample size (n) for each experiment, representing independent biological replicates, specified in the figure legends. The appropriateness of parametric tests was verified by assessing data for normality and homogeneity of variances. Intergroup differences were assessed via unpaired t tests, whereas multigroup comparisons utilized one-way ANOVA with suitable post hoc tests. A significance threshold of *P* < 0.05 was applied throughout the study.

## Results

### HKL ameliorates renal dysfunction and pathological renal injury in diabetic nephropathy model mice

**Fig. 1:**
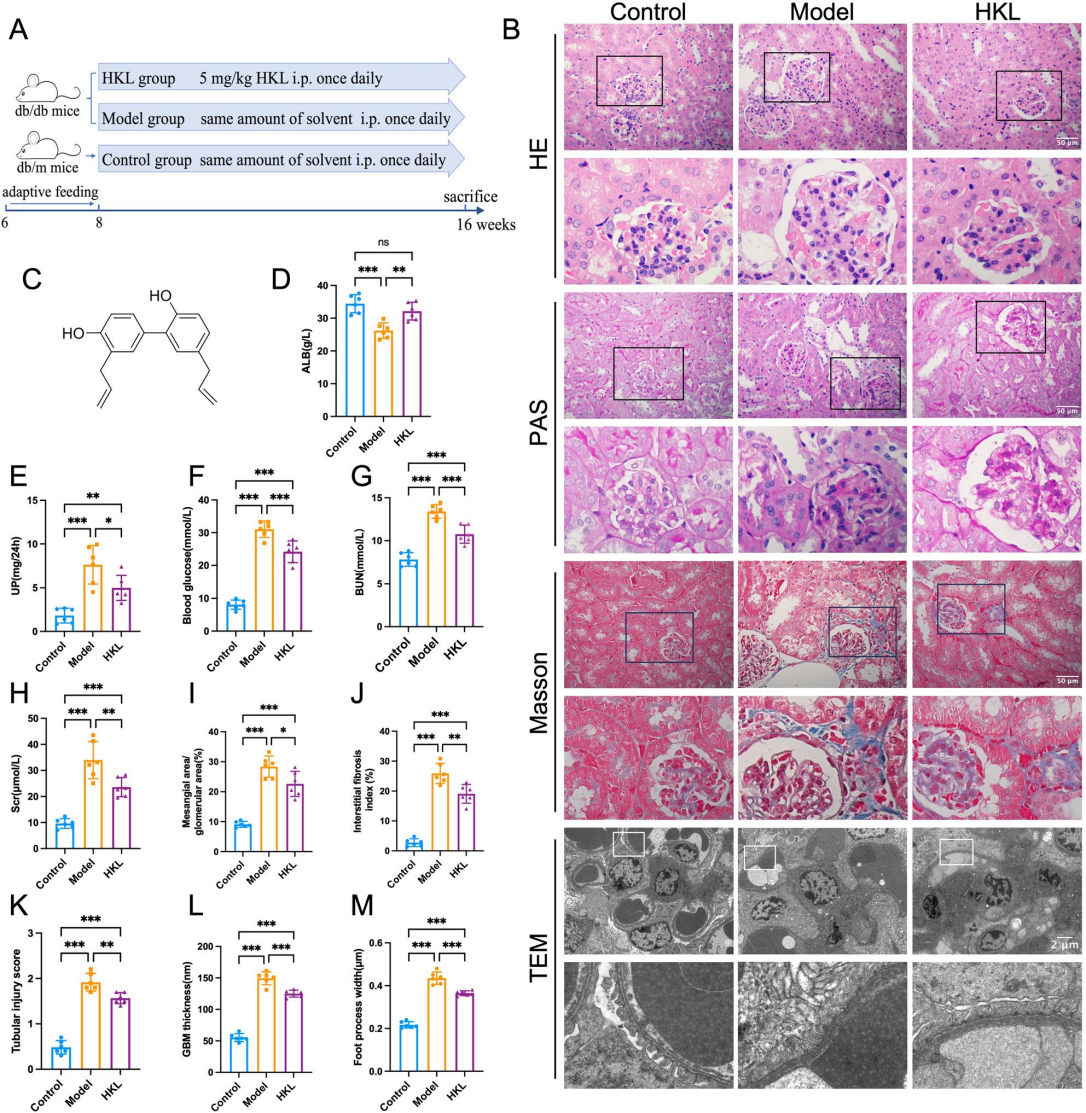
Honokiol ameliorates diabetic nephropathy in db/db mice by improving renal function and histopathology. **A**: Experimental design: Eight-week-old male db/db mice were randomly assigned to the Model group (*n* = 10) or HKL-treated group (50 mg/kg/day, *n* = 10), with age-matched db/m mice used as nondiabetic controls (*n* = 10). All the groups received the indicated interventions for 8 weeks before sacrifice and tissue collection **B**: Representative histological and ultrastructural images: Renal sections stained with HE, PAS, and Masson (scale bar = 50 μm) and TEM micrographs (scale bar = 2 μm) from all experimental groups **C**: Chemical structure of HKL (C_18_H_18_O_2_, molecular weight = 266.33) **D**-H: Biochemical analysis: HKL treatment significantly improved renal function parameters, including reduced albuminuria (**D**), decreased 24 h urine protein excretion (**E**), lowered blood urea nitrogen (**G**), and reduced serum creatinine (**H**) levels compared to the Model group. Blood glucose levels **(F**) were also decreased following HKL administration I‒M: Quantitative histomorphometric analysis: Histograms demonstrate that HKL treatment significantly ameliorated diabetic renal histopathological changes, including reduced mesangial area/glomerular area ratio (**I**), decreased interstitial fibrosis index (**J**), lower tubular injury score (**K**), thinner GBM thickness (**L**), and narrower foot process width (**M**) compared to the Model group The data are presented as the mean ± SD. All data met assumptions for parametric testing. Statistical significance was determined by one-way ANOVA. *P < 0.05, **P < 0.01, ***P < 0.001

Compared with the control group, db/db diabetic mice exhibited significant renal dysfunction, as evidenced by markedly elevated levels of serum creatinine (Scr), blood urea nitrogen (BUN), and 24-hour urinary protein (UP) (*P* < 0.05), along with reduced plasma albumin (ALB) levels (*P* < 0.05). HKL treatment significantly ameliorated these changes in renal function parameters (*P* < 0.05) (Fig. 1D-H), indicating the potent renoprotective effects of HKL in diabetic mice.

Renal histopathological analysis further supported the therapeutic efficacy of HKL. HE, Masson, and PAS staining, as well as TEM, revealed that the control group exhibited normal renal architecture. In contrast, the model group presented characteristic pathological signs of DN, such as glomerular hypertrophy, thickening of the basement membrane, proliferation of mesangial cells and the matrix, fusion of foot processes, vacuolar degeneration of renal tubular epithelial cells, compensatory renal tubular hypertrophy, focal inflammatory cell infiltration, and interstitial fibrosis. Notably, HKL treatment significantly attenuated these pathological alterations (*P* < 0.05) (Fig. 1B, I-M), suggesting its critical role in mitigating renal structural damage in DN.

### HKL inhibits NLRP3/GSDMD-mediated pyroptosis in the kidneys of diabetic nephropathy mice

**Fig. 2:**
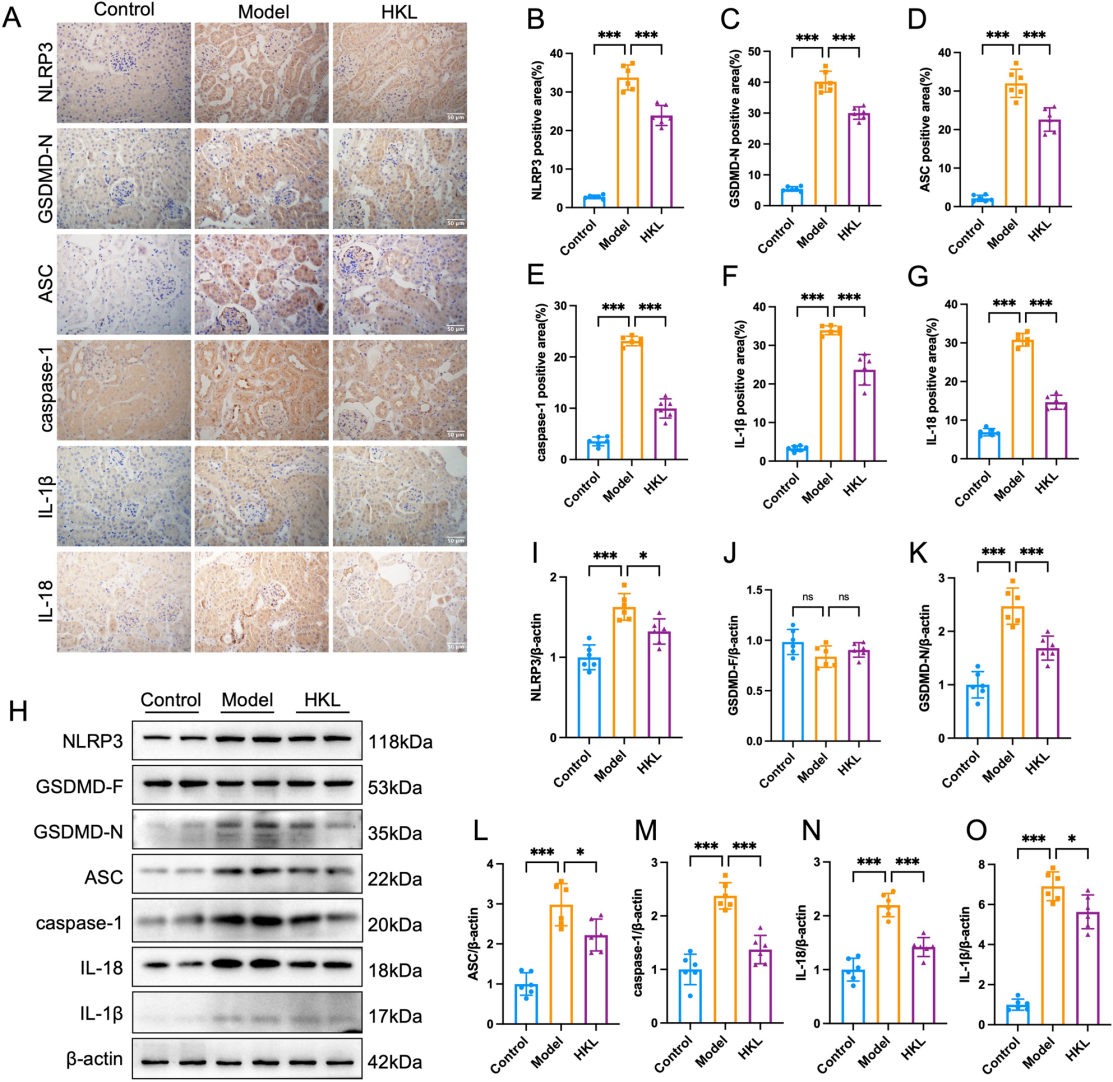
HKL attenuates renal pyroptosis in db/db mice. **A**-**G**: Renal immunohistochemical staining showing the expression levels of NLRP3, GSDMD-N, ASC, caspase-1, IL-1β, and IL-18 in the control, model, and HKL groups (*n* = 6; scale bar = 50 μm). Quantitative analysis demonstrated that db/db mice exhibited significantly enhanced expression of all pyroptosis-related markers compared to control mice, while HKL treatment markedly reduced their expression levels **H**-**O**: Western blot analysis of pyroptosis-related proteins in renal tissues: NLRP3, GSDMD-F, GSDMD-N, ASC, caspase-1, IL-1β, and IL-18 in the renal tissues of the control, model, and HKL groups (*n* = 6). HKL treatment significantly suppressed NLRP3 inflammasome activation and pyroptosis execution, as indicated by reduced GSDMD cleavage and decreased IL-1β and IL-18 levels compared to the model group The data are presented as mean ± SD. All data met assumptions for parametric testing. Statistical significance was determined by one-way ANOVA. *P < 0.05, ***P < 0.001

Immunohistochemical results revealed that compared with the control group, the renal tissues of db/db mice presented significantly upregulated expression of pyroptosis-related marker proteins, including key components of the NLRP3 inflammasome (NLRP3, ASC), its direct activation product (caspase-1), pyroptosis executor protein (GSDMD-N) and its downstream effector molecules (IL-18 and IL-1β) (all *P* < 0.05). After HKL treatment, the upregulation of these pyroptosis-related proteins was markedly suppressed (Fig. 2A-G). Western blot analysis further confirmed these results (*P* < 0.05; Fig. 2H-O). Collectively, these results indicate that HKL might promote renal protection by suppressing the NLRP3/GSDMD-mediated pyroptosis pathway.

### HKL attenuates high glucose-induced pyroptosis in HK-2 cells

**Fig. 3:**
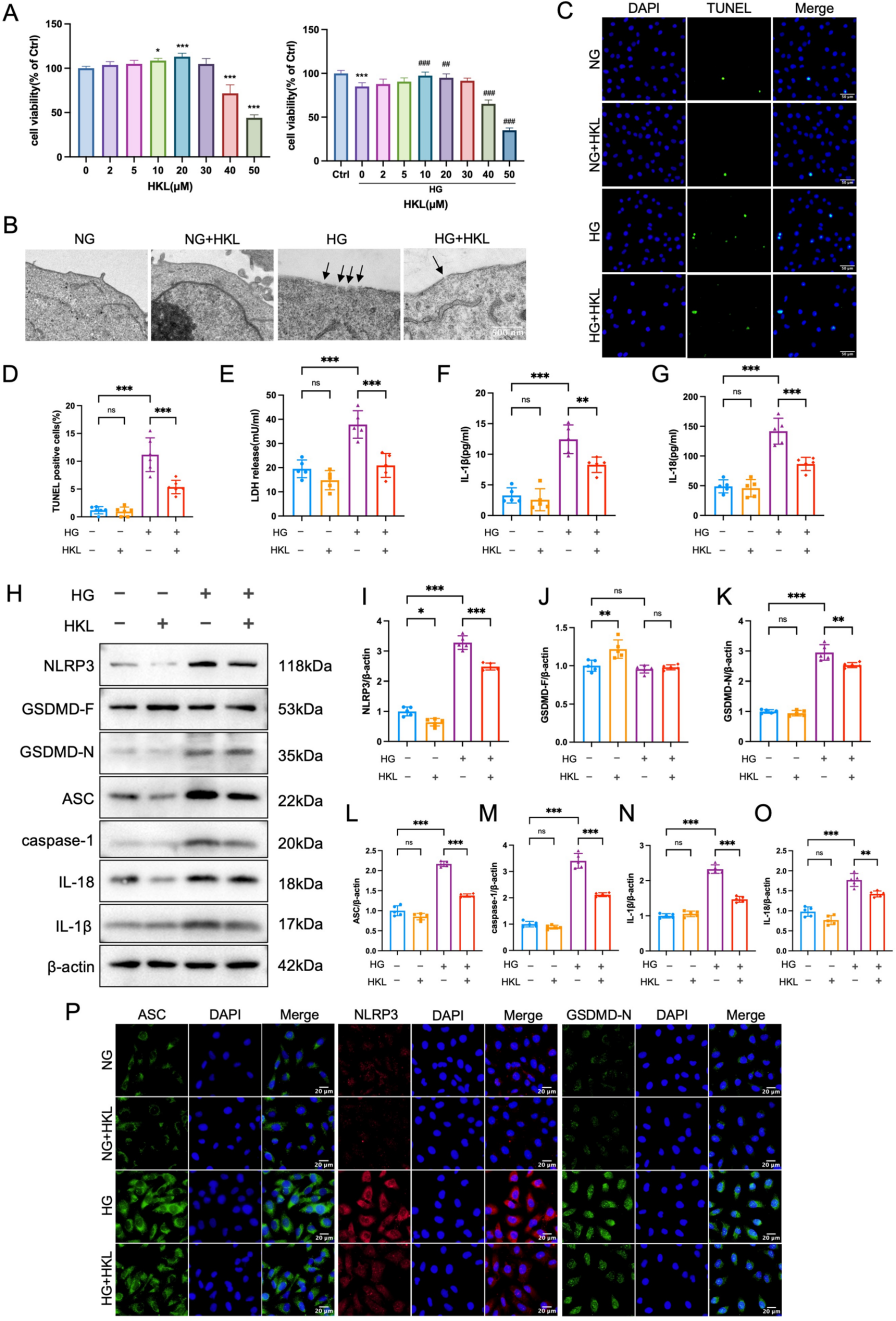
Effect of HKL on high glucose-induced pyroptosis in HK-2 cells. **A**: Cell viability: The effects of different concentrations of HKL on cell viability were detected via a CCK-8 assay (*n* = 6). *P < 0.05 vs. Ctrl, ***P < 0.001 vs. Ctrl, ##*P* < 0.01 vs. HG, ###*P* < 0.001 vs. HG **B**: Representative transmission electron microscopy images: High glucose (HG, 30 mM) induced characteristic membrane rupture, which were markedly attenuated by HKL treatment (10 µM). Normal glucose control (NG, 5.5 mM) and NG + HKL groups showed normal cellular ultrastructure **C**, **D**: TUNEL staining and quantitative analysis: HG stimulation significantly increased TUNEL-positive cells, indicating enhanced pyroptosis, while HKL treatment substantially reduced TUNEL positivity (*n* = 6, scale bar = 50 μm) **E**: LDH release assay (*n* = 6): HKL treatment significantly suppressed HG-induced LDH release, indicating preserved membrane integrity and reduced pyroptosis **F**, **G**: ELISA analysis of inflammatory cytokines (*n* = 6): HKL treatment significantly reduced HG-induced elevation of IL-18 and IL-1β levels in cell culture medium **H**-**O**: Western blot analysis of pyroptosis-related proteins (*n* = 5): Representative blots (**H**) and quantitative analysis (**I**-**O**) demonstrated that HKL treatment significantly inhibited HG-induced activation of pyroptosis **P**: Immunofluorescence analysis: Representative images showing that HKL treatment markedly suppressed HG-induced upregulation and aggregation of ASC, NLRP3, and GSDMD-N (scale bar = 20 μm) The data are presented as mean ± SD. All data met assumptions for parametric testing. Statistical significance was determined by one-way ANOVA. *P < 0.05, **P < 0.01, ***P < 0.001

The results of the CCK-8 assay demonstrated that 10 µM was a safe and effective concentration of HKL for HK-2 cells (Fig. 3A). Under conditions of an elevated glucose concentration (HG, 30 mM), the viability of HK-2 cells significantly decreased (*P* < 0.05), whereas 10 µM HKL markedly restored cell viability (*P* < 0.05).

To evaluate the protective effect of HKL against HG-induced cellular damage, TUNEL staining was performed. The results revealed a significant increase in TUNEL-positive cells in the HG group compared with the control group (*P* < 0.05), whereas the HG + HKL group presented a marked reduction (*P* < 0.05; Fig. 3C, D). Transmission electron microscopy revealed that HG exposure induced characteristic plasma membrane pores, a hallmark of pyroptosis, which were significantly attenuated by HKL treatment (Fig. 3B). Moreover, stimulation with HG led to notable increases in the release of lactate dehydrogenase (LDH) and the levels of the inflammatory cytokines IL-1β and IL-18 (*P* < 0.05). In contrast, treatment with HKL resulted in significant reductions in LDH, IL-18, and IL-1β levels compared with those in the HG group (*P* < 0.05; Fig. 3E-G), indicating the protective effects of HKL against HG-induced cellular damage and the inflammatory response.

Western blot analysis revealed that HG stimulation markedly increased the levels of pyroptosis-related mediators, including NLRP3, ASC, GSDMD-N, cleaved caspase-1, IL-18, and IL-1β (all *P* < 0.05). Notably, HKL treatment significantly downregulated these proteins (*P* < 0.05; Fig. 3H-O), demonstrating its inhibitory effect on HG-induced NLRP3 inflammasome/pyroptosis pathway activation. Immunofluorescence analysis corroborated these findings, revealing increased fluorescence signals of NLRP3, GSDMD-N and ASC in the HG group (*P* < 0.05). These signals were significantly attenuated by HKL treatment (Fig. 3P). The consistent results from both western blot and immunofluorescence analyses strongly suggest that HKL inhibits pyroptosis primarily through targeting the NLRP3 inflammasome pathway.

### HKL confers renal protection in diabetic nephropathy by targeting the SIRT3-NLRP3 axis

**Fig. 4:**
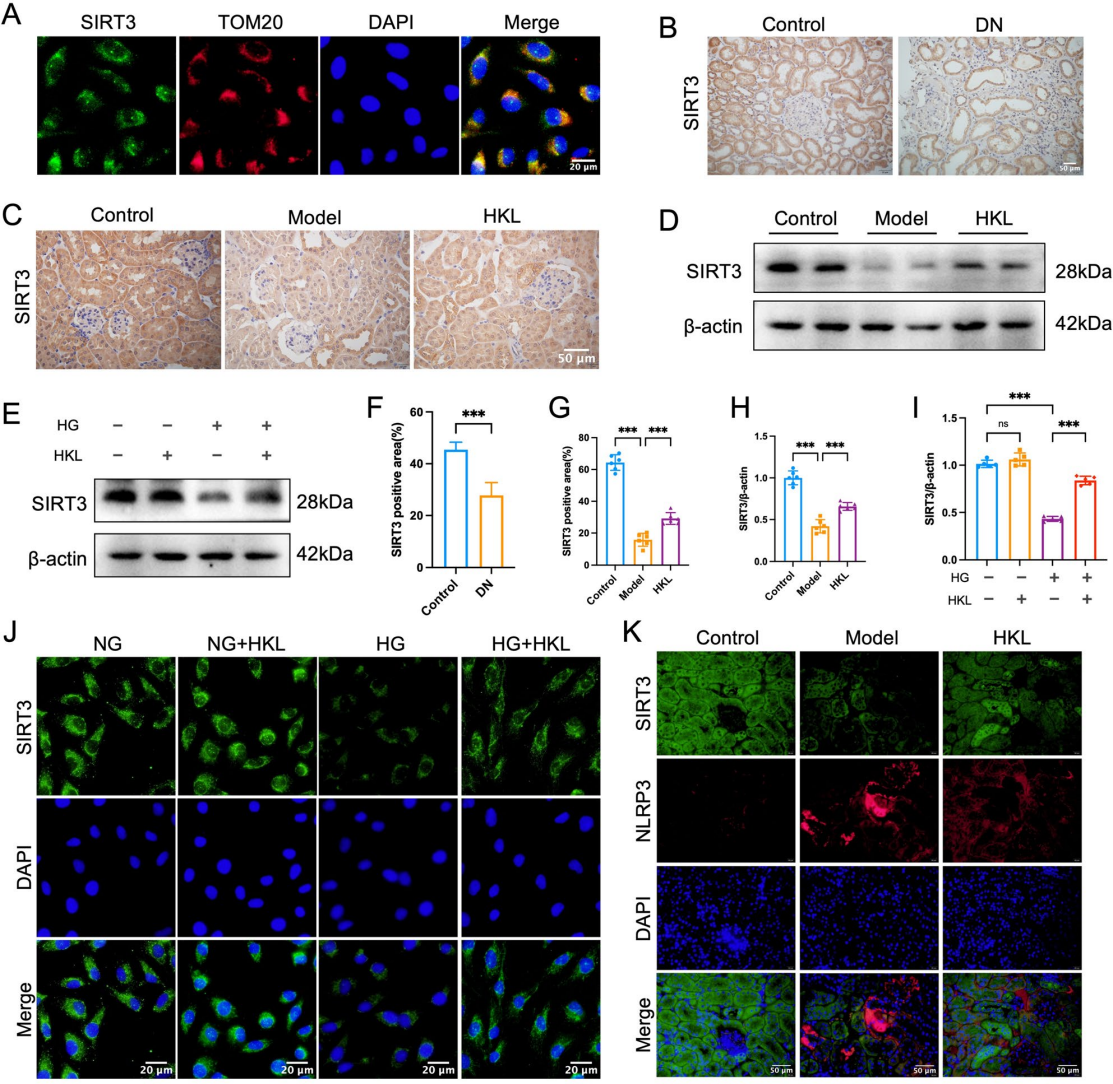
HKL exerts renoprotective effects on high glucose–exposed HK-2 cells and db/db mice through SIRT3. **A**: Immunofluorescence analysis demonstrating mitochondrial localization of SIRT3 in HK-2 cells, shown by colocalization with the mitochondrial marker TOM20 (scale bar = 20 μm) **B**, **F**: Immunohistochemical analysis of SIRT3 expression in human renal tissues: Representative images (**B**) and quantitative data (**F**) (control: *n* = 8 patients; DN: *n* = 8 patients) showing significantly reduced SIRT3 expression in diabetic nephropathy (DN) patients compared to normal kidney tissues (control) (scale bar = 50 μm) **C**, **G**: Renal SIRT3 expression in db/db mice: Immunohistochemical staining (**C**) and quantitative analysis (**G**) (*n* = 6) revealed that HKL treatment significantly restored SIRT3 expression that was reduced in model group mice (scale bar = 50 μm) D, H: Western blot analysis (*n* = 6) confirmed the downregulation of renal SIRT3 protein in db/db mice and its restoration after HKL treatment E, I: SIRT3 protein expression in HK-2 cells: Western blot analysis (*n* = 5) showed that high glucose (HG, 30 mM) significantly decreased SIRT3 expression, while HKL treatment (10 µM) effectively reversed this reduction **J**: Immunofluorescence analysis further confirmed that HKL treatment prevented HG-induced decrease in SIRT3 expression in HK-2 cells (scale bar = 20 μm) K: Double immunofluorescence staining revealed an inverse relationship between SIRT3 (green) and NLRP3 (red) expression in renal tissues, with HKL treatment restoring SIRT3 expression while suppressing NLRP3 inflammasome activation (scale bar = 50 μm) The data are presented as mean ± SD. All data met assumptions for parametric testing. Statistical significance was determined by one-way ANOVA, except for panel F which used unpaired two-tailed Student’s t-test. ***P < 0.001.***P < 0.001

Immunofluorescence analysis demonstrated that SIRT3 was predominantly localized in the mitochondria of HK-2 cells (Fig. 4A), which is consistent with its well-characterized function as a mitochondrial deacetylase. To clarify the function of SIRT3 in pyroptosis, we initially compared SIRT3 expression between renal tissues from DN patients and adjacent normal tissues. These findings indicated a marked decrease in SIRT3 levels among DN patients (*P* < 0.05; Fig. 4B, F).

At the animal model level, both immunohistochemistry and Western blot analyses consistently revealed a marked reduction in SIRT3 expression in the db/db mice compared with the control mice (*P* < 0.05). Notably, HKL treatment significantly restored SIRT3 expression in db/db mice (*P* < 0.05; Fig. 4C, D, G, H). In cellular assays, Western blot and immunofluorescence further confirmed that high glucose stimulation substantially suppressed SIRT3 expression in HK-2 cells, whereas HKL treatment effectively reversed this suppression (*P* < 0.05; Fig. 4E, I, J).

Immunofluorescence costaining analysis revealed that renal tubular areas exhibiting diminished SIRT3 expression consistently presented concomitant increases in NLRP3 levels. HKL treatment coordinately upregulated Sirt3 while downregulating NLRP3 expression (Fig. 4K). Collectively, these results indicate that HKL-mediated attenuation of DN progression is likely mediated through SIRT3 upregulation-associated suppression of NLRP3 inflammasome activation.

### SIRT3 regulates HG-induced pyroptosis and mitochondrial dysfunction

To elucidate the protective mechanisms of SIRT3 in DN, we used SIRT3 overexpression plasmids and specific siRNAs to systematically investigate the regulatory effects of SIRT3 on HG-induced pyroptosis and mitochondrial dysfunction.

**Fig. 5:**
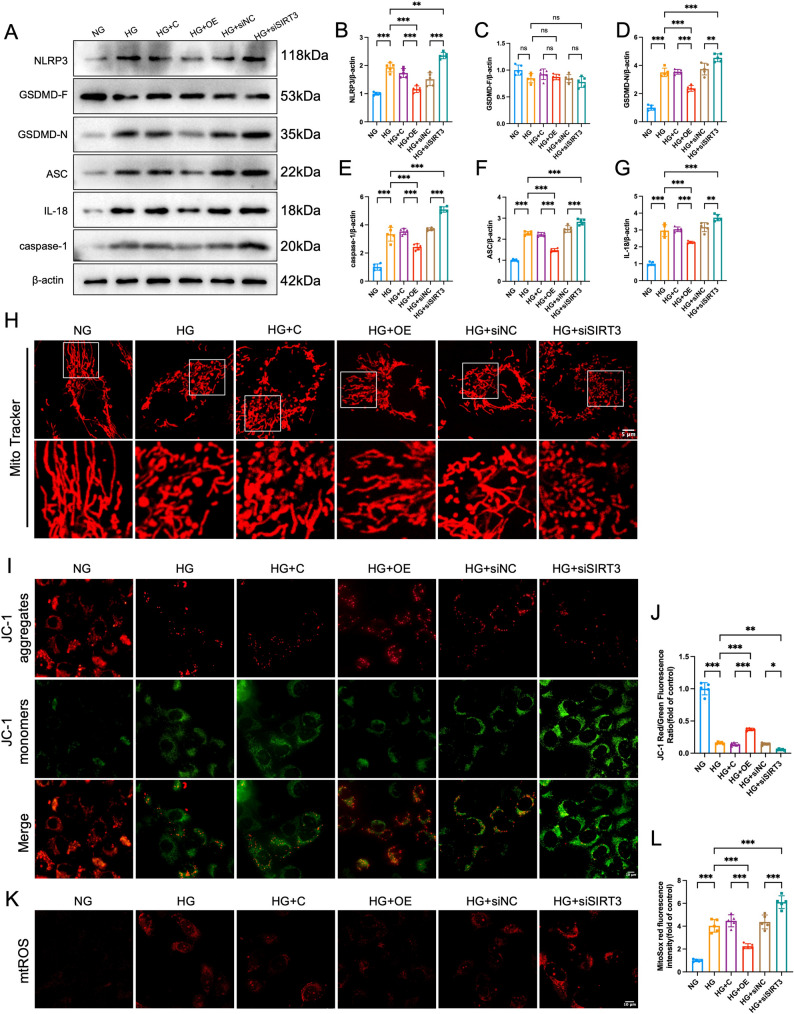
SIRT3 attenuates high glucose-induced mitochondrial dysfunction and pyroptosis. Experimental design: HK-2 cells were divided into six groups: (1) normal glucose control (NG, 5.5 mM), (2) high glucose (HG, 30 mM), (3) HG + empty vector control (HG + C), (4) HG + SIRT3 overexpression (HG + OE), (5) HG + negative siRNA control (HG + siNC), and (6) HG + SIRT3 knockdown (HG + siSIRT3) groups **A**-**G**: Western blot analysis (*n* = 5) showed that high glucose (HG) stimulation significantly upregulated the expression of pyroptosis-related proteins compared to the NG group. This effect was markedly reversed by SIRT3 overexpression, but was further aggravated by SIRT3 knockdown** H**: Representative images of MitoTracker Red staining revealed that HG exposure induced severe mitochondrial fragmentation and network disruption. SIRT3 overexpression ameliorated these morphological abnormalities, while SIRT3 knockdown exacerbated them. (scale bar = 5 μm) **I**, **J**: Assessment of mitochondrial membrane potential (ΔΨm) using JC-1 staining (*n* = 5). The quantitative analysis of the red/green fluorescence ratio (**J**) indicated that HG-induced depolarization of ΔΨm was attenuated by SIRT3 overexpression but was worsened by SIRT3 knockdown (scale bar = 10 μm) K, L: Measurement of mitochondrial ROS production using MitoSOX Red staining (*n* = 5). Quantification of fluorescence intensity (**L**) demonstrated that HG-induced overproduction of mitochondrial ROS was suppressed by SIRT3 overexpression, whereas SIRT3 knockdown led to a further increase (scale bar = 10 μm) Data are presented as mean ± SD. All data met assumptions for parametric testing. Statistical significance was determined by one-way ANOVA. *P < 0.05, **P < 0.01, ***P < 0.001

Western blot analysis revealed that SIRT3 overexpression significantly attenuated the HG-induced upregulation of pyroptosis-related proteins (NLRP3, ASC, cleaved caspase-1, GSDMD-N, IL-1β, and IL-18; *P* < 0.05), whereas SIRT3 knockdown markedly increased their expression (Fig. 5A-G). These results establish SIRT3 as a negative regulator of HG-induced pyroptosis in HK-2 cells.

In mitochondrial functional analysis, Mitotracker staining revealed that HG disrupted mitochondrial network integrity, as manifested by the fragmentation of tubular structures into shortened rods and punctate dots, indicating impaired mitochondrial dynamics. SIRT3 overexpression preserved the reticular mitochondrial architecture, whereas SIRT3 knockdown exacerbated HG-induced mitochondrial fragmentation (Fig. 5H). JC-1 staining confirmed that HG exposure significantly reduced the mitochondrial membrane potential, and this effect was reversed by SIRT3 overexpression but aggravated by SIRT3 knockdown (Fig. 5I, J). Furthermore, MitoSOX staining verified that HG-induced excessive mtROS production was mitigated by SIRT3 overexpression, whereas SIRT3 knockdown further increased mtROS levels (Fig. 5K, L). Collectively, these results provide compelling evidence that SIRT3 plays a pivotal role in maintaining mitochondrial structural integrity and functional homeostasis, thereby regulating mitochondria-derived oxidative stress.

### HKL attenuates pyroptosis via MtROS modulation

**Fig. 6:**
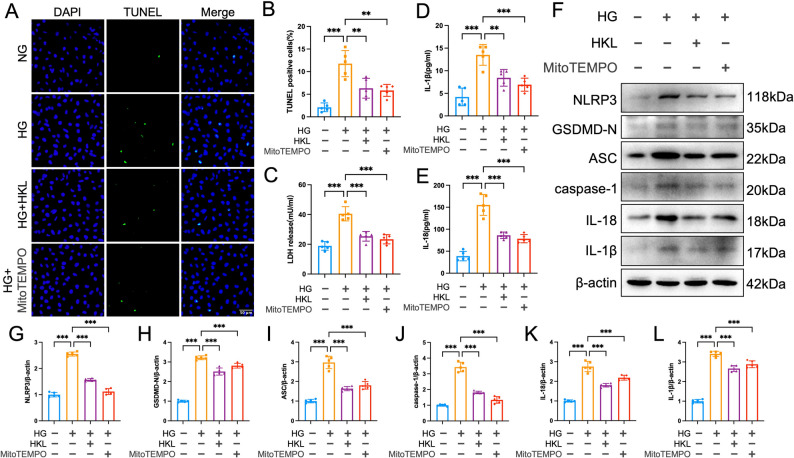
HKL attenuates high glucose-induced pyroptosis through the modulation of mitochondrial ROS signalling. **A**, **B**: Representative TUNEL staining images (**A**) and quantitative analysis (**B**) (*n* = 6) demonstrating that both HKL (10 µM) and the mitochondrial ROS scavenger MitoTEMPO (100 µM) significantly reduced high glucose-induced pyroptotic cell death in HK-2 cells (scale bar = 50 μm) **C**: LDH release assay (*n* = 5): Quantitative analysis showed that HKL and MitoTEMPO treatment similarly attenuated HG-induced LDH release, indicating comparable protective effects against pyroptosis-mediated membrane disruption D, E: ELISA analysis (*n* = 5): Both HKL and MitoTEMPO significantly suppressed HG-induced secretion of IL-1β (**D**) and IL-18 (**E**) into the cell culture medium **F**-**L**: Western blot analysis of pyroptosis-related proteins (*n* = 5): Representative blots (F) and quantitative analysis (**G**-**L**) demonstrated that both HKL and MitoTEMPO effectively inhibited HG-induced pyroptosis pathway activation The data are presented as mean ± SD. All data met assumptions for parametric testing. Statistical significance was determined by one-way ANOVA. *P < 0.05, **P < 0.01, ***P < 0.001

To systematically elucidate the critical role of mitochondrial ROS (mtROS) in the SIRT3-NLRP3 pathway, we employed the mtROS-specific scavenger MitoTEMPO for mechanistic validation. TUNEL staining revealed that both MitoTEMPO and HKL treatment significantly reduced HG-induced cell death (*P* < 0.01; Fig. 6A, B). Consistently, LDH release assays and ELISA demonstrated that MitoTEMPO and HKL effectively suppressed HG-stimulated LDH leakage as well as IL-18 and IL-1β secretion (Fig. 6C-E).

Western blot analysis further confirmed that MitoTEMPO significantly suppressed the increased expression of pyroptosis-related proteins induced by HG. These proteins included NLRP3, cleaved caspase-1, GSDMD-N, ASC, IL-18, and IL-1β, demonstrating effectiveness similar to that of HKL treatment (Fig. 6F-L).

Integrating these findings with our previous SIRT3 expression data, we conclude that HKL likely confers cytoprotection through a sequential mechanism involving SIRT3 upregulation, subsequent inhibition of mtROS overproduction, and ultimately blockade of NLRP3/GSDMD-mediated pyroptosis pathway activation.

### HKL attenuates pyroptosis via the SIRT3‒mtROS axis

**Fig. 7:**
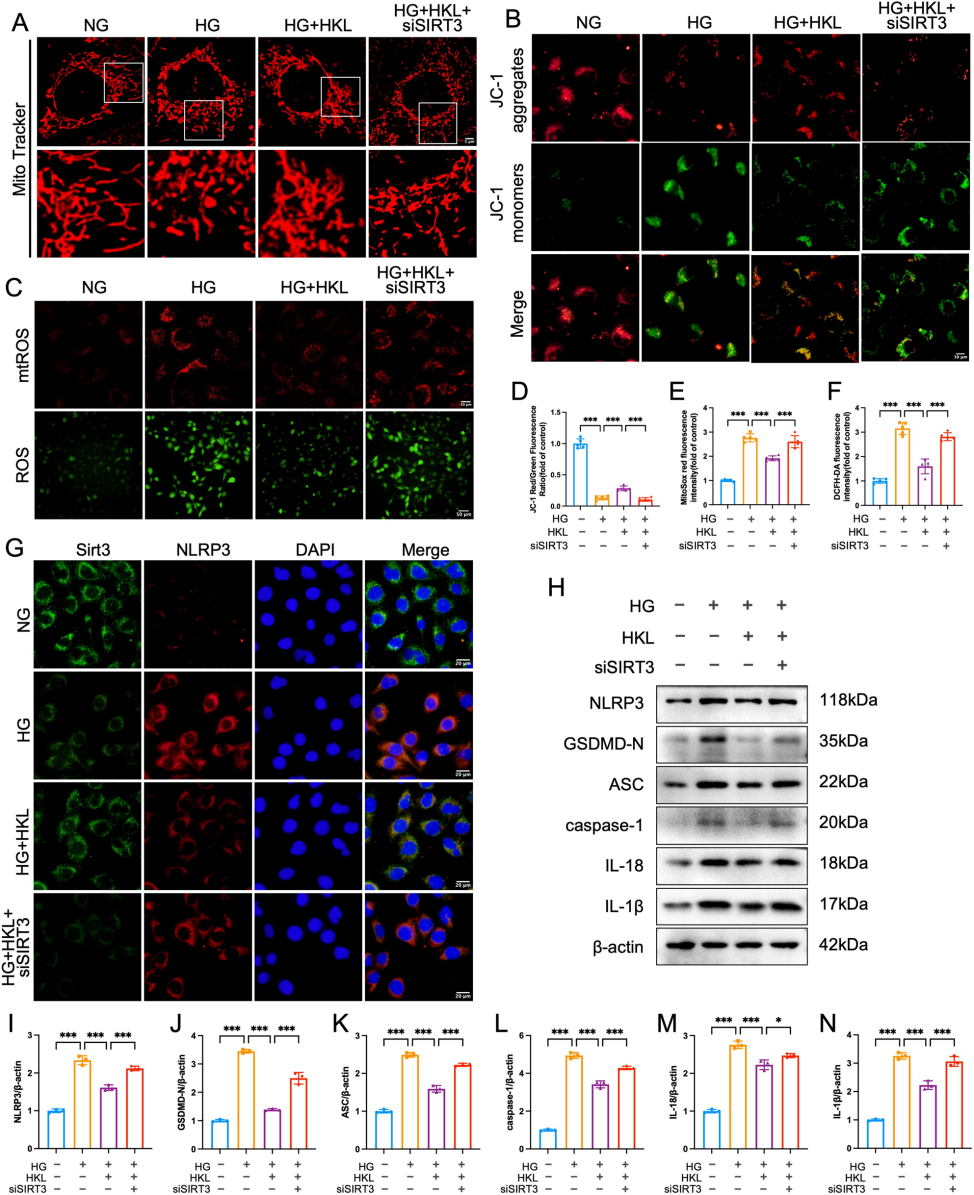
HKL Attenuates High-Glucose-Induced Mitochondrial Dysfunction and Pyroptosis via SIRT3-Dependent Mechanisms **A**: MitoTracker Red staining showing that HKL treatment (10 µM) ameliorated HG-induced mitochondrial fragmentation, an effect abolished by SIRT3 knockdown (scale bar = 5 μm) **B**, **D**: JC-1 staining (*n* = 5) revealed HKL prevented mitochondrial depolarization, which was reversed by SIRT3 knockdown (scale bar = 10 μm) **C**, **E**, **F**: ROS assays (*n* = 5) demonstrated HKL reduced mitochondrial and intracellular ROS, effects attenuated by SIRT3 knockdown. (scale bar = 10/50 µm) G: Immunofluorescence analysis revealed HKL-enhanced SIRT3 expression with concurrent NLRP3 suppression, effects that were reversed by SIRT3 knockdown (scale bar = 20 μm) H‒N: Western blot analysis (*n* = 3) demonstrated HKL-mediated suppression of pyroptosis-related proteins (NLRP3, ASC, caspase-1, GSDMD-N, IL-1β, IL-18), which was abolished by SIRT3 knockdown The data are presented as mean ± SD. All data met assumptions for parametric testing. Statistical significance was determined by one-way ANOVA. *P < 0.05, **P < 0.01, ***P < 0.001

To determine whether the renoprotective effects of HKL specifically depend on the SIRT3-mtROS pathway, we established SIRT3-knockdown HK-2 cells for functional validation. HG stimulation not only induced mitochondrial fragmentation from elongated filaments to shortened rods but also significantly decreased the JC-1 red to green fluorescence ratio (*P* < 0.01), indicating substantial impairment of mitochondrial structure and function. Although HKL treatment effectively ameliorated these pathological alterations, its protective effects were markedly attenuated under SIRT3-knockdown conditions (Fig. 7A, B, D).

Using DCFH-DA and MitoSOX probes, we found that HKL significantly reduced HG-induced ROS accumulation and that this antioxidant capacity was also SIRT3 dependent (Fig. 7C, E, F). Immunofluorescence colocalization analysis further demonstrated that HKL not only restored HG-induced suppression of SIRT3 expression but also downregulated NLRP3 overexpression; however, these regulatory effects of HKL were substantially diminished after SIRT3 knockdown (Fig. 7G).

At the molecular level, Western blot analysis confirmed that SIRT3 knockdown significantly attenuated the inhibitory effects of HKL on proteins associated with pyroptosis (NLRP3, ASC, GSDMD-N, cleaved caspase-1, IL-1β, and IL-18; *P* < 0.05; Fig. 7H-N). Taken together, these results provide compelling evidence that the SIRT3-mtROS axis serves as the key pathway through which HKL suppresses NLRP3 inflammasome-dependent pyroptosis to exert its renoprotective effects.

## Discussion

DN represents one of the most prevalent microvascular complications of diabetes and a primary contributing factor to ESRD worldwide, stemming from hyperglycemia-induced metabolic dysregulation that progressively damages renal cells, including tubular epithelial cells. Accumulating evidence implicates pyroptosis, which is characterized by cellular swelling, membrane perforation, and proinflammatory mediator secretion, as an important driver of DN progression [28–30].

Pyroptosis‌ is a programmed cell death pathway that is mediated by inflammasomes and executed by gasdermin family proteins [31]. This process initiates when pattern recognition receptors identify microbial or endogenous danger signals, promoting NLRP3 inflammasome assembly and caspase-1 activation. The enzymatically active caspase-1 cleaves GSDMD to liberate its N-terminal domain (GSDMD-N), which subsequently oligomerizes and forms transmembrane pores in the plasma membrane. GSDMD-N oligomerization generates plasma membrane pores that disrupt osmotic balance, causing cellular swelling and eventual membrane rupture. This lytic process facilitates the release of inflammatory mediators, amplifying immune responses [32]. In support of the clinical relevance of this pathway, renal biopsies from DN patients exhibit marked upregulation of NLRP3 and GSDMD compared with those from healthy controls [33]. Consistent with these findings, in vitro studies have consistently demonstrated increased expression of NLRP3, caspase-1, GSDMD-N, IL-18, and IL-1β in HK-2 cells exposed to high-glucose conditions [34]. These observations are fully congruent with the results of our experiments in both animal models and cultured renal cells.

The high metabolic demand of the kidney necessitates an extensive mitochondrial network to sustain glomerular filtration and tubular reabsorption. As the cellular powerhouses and primary sources of ROS, mitochondria are critically involved in renal homeostasis. When mitochondrial function becomes dysregulated, the resulting energy crisis and oxidative stress trigger multiple cell death pathways, including pyroptosis [35]. Mitochondrial ROS (mtROS) play a dual role in cellular physiology: in addition to mediating oxidative damage, they function as critical signalling molecules that regulate cellular processes. When mtROS accumulation surpasses physiological levels, oxidative modification promotes the oligomerization of critical NLRP3 inflammasome components, thereby activating the caspase-1/GSDMD-dependent pyroptotic pathway. This mechanism contributes substantially to various metabolic disorders, highlighting mtROS-mediated pyroptosis as a promising therapeutic target [36–38].

SIRT3, a key NAD+-dependent deacetylase, serves as a crucial regulator of mitochondrial homeostasis by modulating protein acetylation states [39]. It enhances cellular antioxidant defenses through SOD2 activation [40–42] and fine-tunes mitochondrial dynamics via regulation of fission and fusion proteins [43]. These functions underlie SIRT3’s renoprotective effects, which include mitigating oxidative stress, suppressing fibrosis, and modulating inflammation in various renal pathologies, particularly DN [18, 44–46].

Current research has identified several SIRT3 modulators that show promise in kidney injury models [47, 48]. Among these, honokiol (HKL) has emerged as a compound that potently upregulates SIRT3 and ameliorates DN through multiple mechanisms, including alleviating endoplasmic reticulum stress and inhibiting Rho kinase/ROCK signalling [49, 50]. While previous studies have established the importance of SIRT3 activation in DN management, our study provides the first demonstration that HKL’s nephroprotective effects are specifically mediated through SIRT3-dependent suppression of NLRP3 inflammasome signalling and GSDMD-mediated pyroptosis. This novel finding not only confirms previous reports on HKL’s renoprotective effects but also establishes an innovative link between mitochondrial quality control and inflammasome regulation in DN pathogenesis.

Through systematic investigation, we elucidated how HKL ameliorates DN via modulation of the SIRT3-mtROS-NLRP3 signalling axis. In db/db mice, HKL administration significantly improved renal function and attenuated characteristic renal lesions. At the molecular level, HKL enhanced mitochondrial homeostasis through SIRT3 upregulation, as evidenced by preservation of the mitochondrial network architecture, restoration of the membrane potential, and a substantial reduction in mtROS accumulation. Importantly, HKL suppressed NLRP3 inflammasome activation in a SIRT3-dependent manner, as indicated by decreased GSDMD-N cleavage and reduced IL-1β/IL-18 secretion. Genetic validation experiments confirmed the essential role of SIRT3, with knockdown models showing attenuated HKL efficacy, while the mtROS scavenger MitoTEMPO replicated the therapeutic effects of HKL. These findings establish the SIRT3-mtROS axis as a key regulatory pathway in DN and provide a molecular foundation for novel pyroptosis-targeting therapies.

As the first study to delineate HKL’s mechanism through the regulation of mtROS and pyroptosis, our work not only advances the understanding of mitochondrial-immune crosstalk in DN but also highlights HKL’s translational potential as a next-generation therapeutic agent. Unlike current first-line therapies such as RAS or SGLT2 inhibitors that primarily modulate hemodynamics and show limited efficacy in advanced chronic kidney disease [51], HKL directly targets core pathological mechanisms including mitochondrial dysfunction and inflammatory activation. Its multimodal mechanism combines potent antioxidant and anti-inflammatory properties with a favorable preclinical safety profile, positioning HKL as a promising candidate for either standalone or adjunctive therapy in advanced DN cases where existing treatments remain inadequate.

While the present findings indicate the therapeutic potential of HKL, several limitations must be acknowledged to guide future research. First, while the renoprotective effects of HKL have been demonstrated in db/db mouse models and in vitro systems, further validation in more translationally relevant models is warranted to better address the pathological heterogeneity of human DN. Additionally, key pharmacological properties of HKL—such as its bioavailability, optimal dosing regimens, safety profile, long-term toxicity in humans—require further elucidation to support future clinical translation. These aspects should be systematically investigated through well-designed pharmacokinetic and pharmacodynamic studies. Finally, the potential synergistic effects between HKL and established pharmacotherapies, including SGLT2 inhibitors and RAS inhibitors, warrant systematic investigation. These studies may reveal novel combination therapies with enhanced clinical efficacy.

## Conclusion

Overall, this study elucidates a novel molecular mechanism whereby HKL confers renal protection through the modulation of the SIRT3-mtROS-NLRP3 axis. More significantly, these findings establish a crucial theoretical framework for developing innovative therapeutic strategies against DN that target the mitochondrial-inflammatory network.

## Supplementary Information

Supplementary Material 1:Structural characterization of honokiol by ¹H NMR spectroscopy and UPLC‒MS analyses. A: ¹H NMR spectrum in CDCl₃ showing diagnostic proton signals. B: UPLC chromatogram obtained with a C18 column using acetonitrile/water gradient elution. C: ESI mass spectrum confirming the molecular weight

## Data Availability

All the data generated during the study can be obtained upon reasonable request from the corresponding author.
