# Supplementary figures and images for: Honokiol attenuates diabetic nephropathy by targeting SIRT3 to suppress mitochondrial ROS-induced pyroptosis
Source: Diabetol Metab Syndr. 2025 Dec 2;18:7. doi: 10.1186/s13098-025-02045-4 (PMC12777499; doi:10.1186/s13098-025-02045-4)

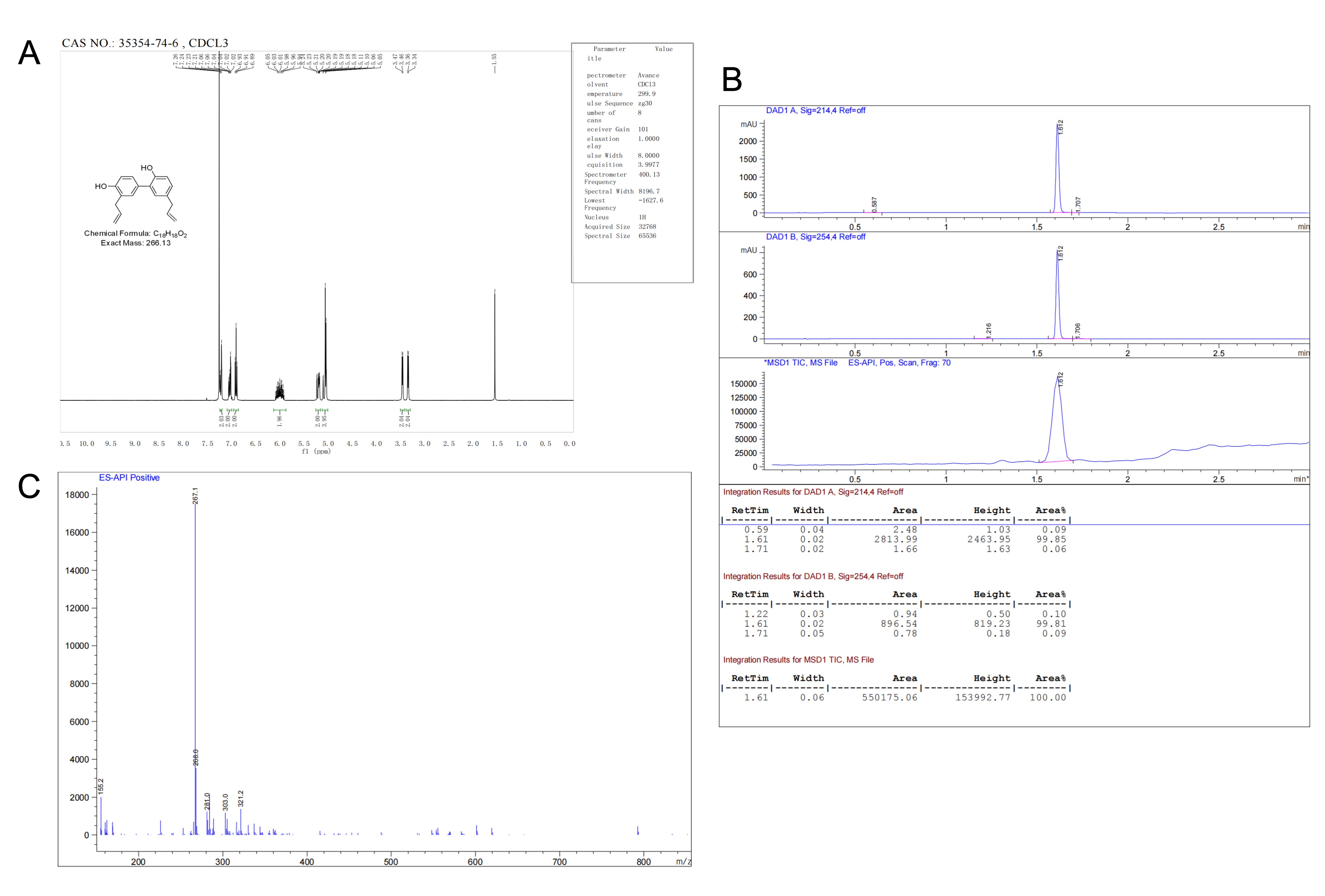

Supplement: Supplementary file 1 — Supplementary Material 1:Structural characterization of honokiol by ¹H NMR spectroscopy and UPLC‒MS analyses. A: ¹H NMR spectrum in CDCl₃ showing diagnostic proton signals. B: UPLC chromatogram obtained with a C18 column using acetonitrile/water gradient elution. C: ESI mass spectrum confirming the molecular weight [file 13098_2025_2045_MOESM1_ESM.jpg]
